# Editorial: *Acta Biochimica Polonica* reviews | 2023–2024

**DOI:** 10.3389/abp.2025.15744

**Published:** 2025-11-07

**Authors:** Michał Rurek, Jarosław Czyż, Zbigniew Heleniak, Paulina Niedźwiedzka-Rystwej, Witold N. Nowak, Milena Paw, Dawid Wnuk

**Affiliations:** 1 Department of Molecular and Cellular Biology, Institute of Molecular Biology and Biotechnology, Faculty of Biology, Adam Mickiewicz University, Poznań, Poland; 2 Department of Cell Biology, Faculty of Biochemistry, Biophysics and Biotechnology, Jagiellonian University in Kraków, Kraków, Poland; 3 Department of Nephrology, Transplantology and Internal Medicine, Medical University of Gdańsk, Gdańsk, Poland; 4 Institute of Biology, University of Szczecin, Szczecin, Poland; 5 August Chełkowski Institute of Physics, Faculty of Science and Technology, University of Silesia in Katowice, Chorzów, Poland

**Keywords:** cardiovascular health, microbiota, mitochondria, potassium chennels, plant transcriptome

The aim of the Special Issue “*Acta Biochimica Polonica* Reviews | 2023–2024” published in *Acta Biochimica Polonica* was to gather review articles from various research areas, focusing on cellular and molecular biology, and biomedicine. This editorial paper provides a concise overview of four publications published within the frames of this Special Issue.

Plant transcriptomes are highly complex entities shaped by a multitude of stressors in a spatiotemporally heterogeneous manner (Imadi et al., 2015). Numerous abiotic stressors represent part of the normal ecological niche to which plants are adapted, often triggering constitutive or primed stress-response programs. Two papers published in the Special Issue, the first (Rurek) and the third (Rurek and Smolibowski), provide a deepened meta-analysis of differences in plant transcriptomes under the impact of various stressors and across diverse vegetative and generative organs and tissues. Data were taken from numerous model, crop and medicinal plant species, and from various transcriptomic platforms, including single cell and spatial RNA-seq (Peirats-Llobet et al., 2023; Wang et al., 2024; Guo et al., 2025). The enriched gene ontology terms from affected gene families and the respective transcription factors were compared. The supplementary data in both papers provide summary of quantitative alterations in gene expression patterns and details on experimental conditions. In their review, Rurek and Smolibowski compared dynamic transcriptomic responses to selected abiotic and biotic stressors (Wang et al., 2020). Stress responses involved both species/stressor-specific but also shared gene families (coding mostly photosynthetic proteins and secondary metabolism enzymes). In turn, Rurek aimed to characterize the most relevant spatiotemporal transcriptomic responses in selected vegetative and generative plant organs and tissues in the developmental context (Huang et al., 2016). The dynamicity of organ/tissue-specific responses was also compared in the context of the complexity and diversity of recently characterized plant genomes and transcriptomes. The specificity of organ/tissue-specific transcriptomes related to the over-representation of certain gene families and transcription factors was also emphasized. In addition, the perspectives for the application of medicinal plant species in industrial synthesis of valuable secondary metabolites were outlined based on organ-specific transcriptomic responses (Guo et al., 2021).

From the reviews by Rurek and Smolibowski and Rurek, the relevance of current plant transcriptomic studies both for basic research as well as for agriculture can be visioned. Functional analyzes would allow for better characterization of stress-responsive genes and less-known stress acclimatory mechanisms. On this basis, more stress-resistant crop cultivars could be designed in the future, based on advanced tools of plant biotechnology (Sriboon et al., 2020; Liu et al., 2024). Such actions should also benefit more from plant metabolome heterogeneity. More consistent usage of the potential of medicinal species in the synthesis of pharmaceutically important compounds may help to combat a range of lifestyle-related diseases.

Modulation of the gut microbiome is another promising option to deal with the fatal increase in lifestyle-related diseases. The second paper in the Special Issue (Gofron et al.) provides the meta-analysis of the complex effects of *Akkermansia muciniphila* (*A. municiphila*) on human health and their therapeutic applications. This facultatively anaerobic, gram-negative microbial species, initially isolated in 2004, colonizes the mucosa of the colon and rectum crypts and specializes in mucin degradation. Intensity of crypt colonisation by *A. muciniphila* correlates with the increased likelihood of colorectal cancer (Derrien et al., 2010; Nath and Mukherjee, 2014). However, available data also indicate the participation of *A. municiphila* in cobalamin biogenesis and in the maintenance of intestinal and cardiovascular health; these bacteria hamper endothelium inflammation and atherosclerosis (Shin et al., 2014; Li et al., 2016; Mok et al., 2020). By various cellular and molecular mechanisms, they control body weight and cardiovascular disorders, including atrial fibrillation, and can be used in obesity therapy (Dao et al., 2016; Depommier et al., 2019; Luo et al., 2022). *A. municiphila* also participates in the biogenesis of the intestinal mucosa layer and in the host protection against several inflammatory diseases (Qu et al., 2021). However, because of the limited number of reports, the interconnections between these activities and their general effects on human welfare need further exploration. In addition, the safety and potential of consuming living cultures of *A. municiphila* to maintain their positive cardiovascular effects, as well as more specialized studies on the impact of *A. municiphila* on the circulatory system should be investigated.

For a long time, mitochondria were mainly regarded as the centers of bioenergetical processes and cellular metabolism. Novel aspects of mitochondria function include their participation in cytoprotection, cellular senescence, tumorigenesis, fibrosis and inflammatory responses (Li et al., 2020; Barrera et al., 2021; Chapa-Dubocq et al., 2023; Szabo and Szewczyk, 2023). The fourth publication of this Issue, a mini-review by Szewczyk, focuses on recent findings concerning the role of mitochondrial K-dependent (mitoK) channels in the regulation of mitochondrial and cellular physiology. The discovery of mitoK channels in 1991 (Inoue et al., 1991), although initially underestimated, led to their recognition as crucial players in multiple aspects of mitochondrial biogenesis across various taxa (Koszela-Piotrowska et al., 2009; Laskowski et al., 2015; Szabo and Szewczyk, 2023). In this paper, the research of multiple mitochondrial K-dependent channels were first summarized in historical context. Next, fundamental observations and most important ideas on their biological significance were discussed, with the emphasis on the regulation of reactive oxygen species (ROS) generation, membrane potential, respiration and the integrity of the mitochondrial inner membrane (Bednarczyk et al., 2013; Kulawiak et al., 2023). Moreover, the role of mitoK channels in cardio/neuroprotection and inflammation was stressed along with their function as the regulators of the cell fate (Liu et al., 1999; Garlid, 2000; Garlid et al., 2003; Busija et al., 2004; Leanza et al., 2014; Barrera et al., 2021). Broad activities of mitoK channels discovered so far indicate that the development of specific mitoK channel-specific inhibitors, new approaches for *in situ* monitoring of mitoK channel activity, and new routes for broadening our knowledge on molecular identity of mitoK channels would be helpful for their application as targets in medicine.

In conclusion, these four studies collectively provide valuable insights into various aspects of molecular, cellular biology as well as biomedicine. Although the spectrum of topics discussed in these papers is relatively broad, they emphasize the need for more deepened studies involving advanced methodological solutions to gain better insights into investigated biological processes and their regulation. These contributions underscore the growing importance of integrating molecular and physiological data. The translational potential of such research is evident: organ-specific plant transcriptomics offers opportunities for optimized production of bioactive compounds, *A. muciniphila* emerges as a promising microbiome-based tool for metabolic and cardiovascular disease prevention, and mitoK channels represent novel therapeutic targets in cardio- and neuroprotection. Such integrative and interdisciplinary approaches will not only deepen our mechanistic understanding, but also open new avenues for translational applications in biotechnology and medicine.
